# Identification of Key Bioactive Compounds of Medicine–Food Homologous Substances and Their Multi-Target Intervention Effects in Osteosarcoma Treatment

**DOI:** 10.3390/ijms27031360

**Published:** 2026-01-29

**Authors:** Jie Ren, Xue Zhang, Siyu Chen, Ruiming Liu, Pengcheng Yi, Shuang Liu

**Affiliations:** School of Basic Medical Sciences, Jiamusi University, Jiamusi 154007, China

**Keywords:** osteosarcoma, medicine–food homologous substance, bioactive compounds, molecular docking, molecular dynamics simulation, functional enrichment

## Abstract

Osteosarcoma (OS), a highly aggressive bone malignancy, is hard to treat due to complex molecular mechanisms. This study aimed to identify key bioactive compounds from medicine–food homologous (MFH) substances for OS intervention. We analyzed GEO transcriptomic data to get 317 differentially expressed genes (DEGs), screened bioactive compounds from 106 MFH via dual databases, predicted compound–DEG protein interactions with GraphBAN, and filtered 11 core compounds through drug-likeness/toxicity evaluations. Regulatory networks identified 5 key target genes (SOST, ACACB, TACR1, GRIN2B, MPO), 10 key compounds (e.g., ellagic acid dihydrate) and 8 MFHs (e.g., Daidaihua). Molecular docking/MD confirmed stable complexes. GSEA/GSVA revealed pathway dysregulation (e.g., upregulated WNT signaling), and immune analysis showed altered infiltration of 5 cell subsets. 143B cell experiments and qRT-PCR validated findings. MFH-derived compounds, especially ellagic acid dihydrate, have multi-target anti-OS potential, laying a foundation for novel OS therapeutics.

## 1. Introduction

Osteosarcoma (OS), the most common type of primary malignant bone tumor, primarily affects adolescents and children [1,2,3]. Characterized by high invasiveness, early lung metastasis, and significant recurrence rates, the current clinical treatment standard combines surgical resection with neoadjuvant and adjuvant chemotherapy [4,5]. Although multi-drug regimens have substantially improved overall survival rates over decades, patients with metastatic or recurrent tumors still face a five-year survival rate below 30% [6,7,8,9,10]. Furthermore, existing chemotherapeutic agents frequently exhibit strong drug resistance and severe side effects, severely compromising treatment efficacy and quality of life [11,12,13,14]. Consequently, developing new low-toxicity, highly effective therapeutic strategies has become an urgent priority in OS treatment. Genomic research is crucial for understanding the molecular mechanisms of OS, revealing key genes and signaling pathways involved in tumor development, metastasis, and drug resistance. These insights will provide a theoretical foundation for targeted therapies [15,16,17]. Through precise gene regulation, multi-target intervention strategies can be developed, which will help overcome the limitations of traditional treatment methods, especially in dealing with the recurrence and metastasis of OS, and open up new therapeutic ideas.

Medicinal and food homologous (MFH) substances are natural resources that can be used both as ingredients and medicinal materials, characterized by high safety, minimal toxicity, and dual nutritional and pharmacological value [18,19,20]. Recent studies have shown that active components in MFH (such as flavonoids, polyphenols, polysaccharides, and alkaloids exhibit significant effects in inhibiting tumor cell proliferation, inducing apoptosis, suppressing metastasis, and enhancing immune responses [21,22,23,24]. For instance, certain flavonoid compounds exert anti-oxygen-sensing effects by regulating signaling pathways like PI3K/Akt and MAPK [25,26,27]. Additionally, active components from hawthorn have demonstrated cancer-inhibiting capabilities through Wnt/β-catenin, AMPK/mTOR, and NF-κB/EMT pathways in vitro [28]. Compared to traditional chemotherapy drugs, MFH active components often target multiple biological mechanisms, enabling comprehensive regulation across various therapeutic pathways. This makes them promising candidates for the prevention and treatment of malignant tumors such as OS [18,28]. However, specialized research on the direct intervention of medicinal food homology substances and their active ingredients against osteosarcoma remains relatively scarce. Consequently, their specific targets and molecular mechanisms of action remain elusive. Therefore, systematic mining and identification of key active ingredients in MFH substances and in-depth analysis of their mechanisms of action are of great significance for the development of new natural anti-OS drugs and the expansion of clinical application of MFH resources.

**Figure 1:**
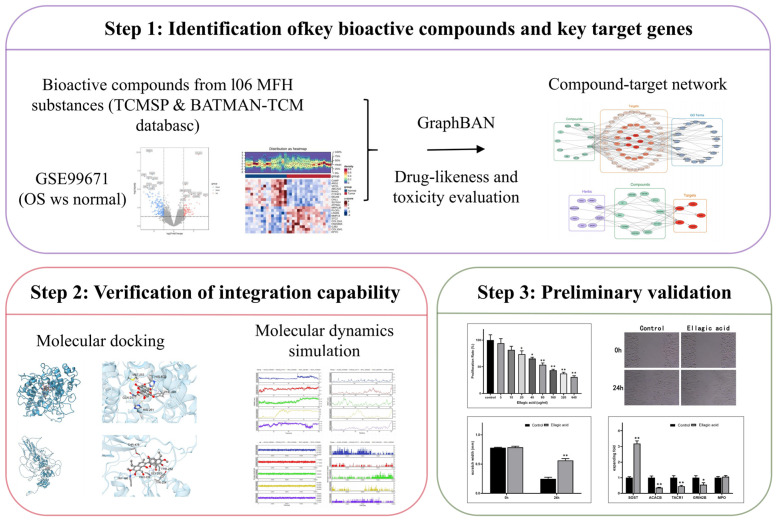
This design diagram illustrates the workflow of integrating network pharmacology and molecular docking, culminating in in vitro experimental validation of the therapeutic efficacy of MFH in treating OS. “(In Step 3, * *p* < 0.05; ** *p* < 0.01)”.

To investigate the multi-target mechanisms of MFH substances in combating OS, this study adopts an integrated strategy of computational biology and experimental validation for systematic analysis. First, we will construct a compound-target interaction network by integrating OS transcriptomic data with a library of MFH active ingredients, thereby screening for core active components and key target genes. Subsequently, the stability of core interactions will be assessed through molecular docking and dynamics simulations, while the underlying biological mechanisms will be elucidated via functional enrichment and immune infiltration analyses. Finally, key predictions will be experimentally validated through in vitro cell experiments. This comprehensive research will not only provide a systematic pharmacological basis for the anti-OS effects of MFH substances but also lay a solid foundation for developing low-toxicity, high-efficacy, multi-target natural drugs for OS. The research protocol is illustrated in Figure 1.

## 2. Results

### 2.1. Obtaining and Analyzing the Transcriptome DEGs

**Figure 2:**
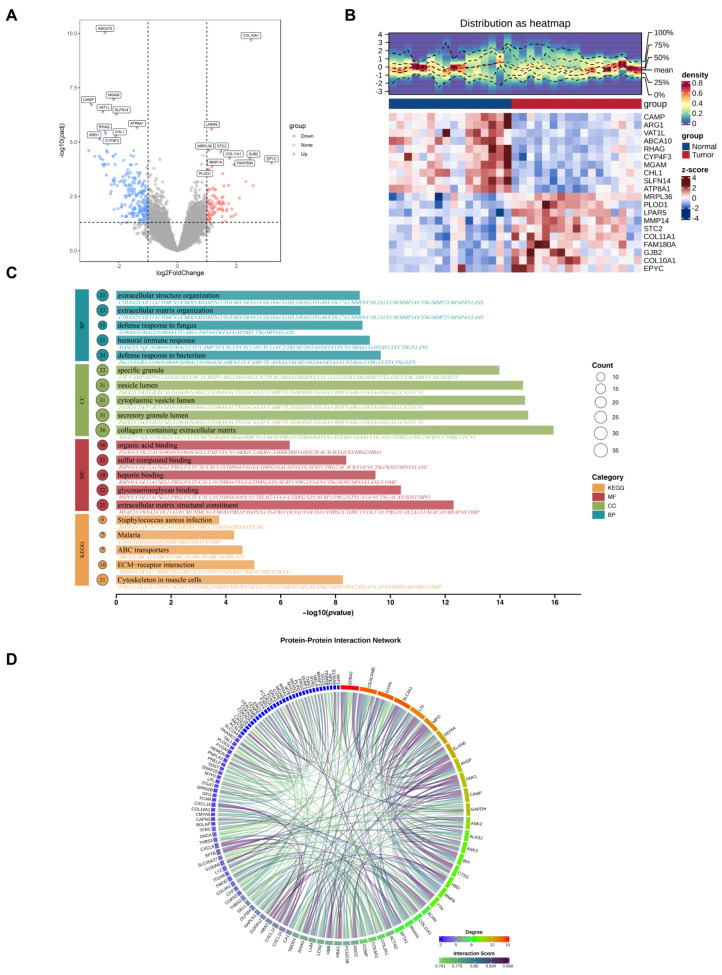
Identification of Osteosarcomas-related targets. (**A**) Volcano plot. (**B**) Heatmap of the 317 most significant DEGs. (**C**) Bar plot from GO enrichment analysis. (**D**) Visualization of cross-target PPI network.

When analyzing the GSE99671 dataset, we identified a total of 317 significant DEGs between the OS and normal groups after strict screening criteria, with 96 being upregulated and 221 being downregulated in OS samples (Figure 2A,B). GO enrichment analysis yielded 1097 significant terms (*p* < 0.05), comprising 899 BPs, 92 CCs, and 106 MFs (Supplementary Table S1). In the BP category, candidate genes were significantly enriched in defense response to bacterium (GO:0042742), humoral immune response (GO:0006959), defense response to fungus (GO:0050832), extracellular matrix organization (GO:0030198), and extracellular structure organization (GO:0043062) (Figure 2C). For the cellular component (CC) category, we observed enrichment in several key terms: collagen-containing extracellular matrix (GO:0062023), secretory granule lumen (GO:0034774), cytoplasmic vesicle lumen (GO:0060205), vesicle lumen (GO:0031983), and specific granule (GO:0042581). After conducting GO enrichment analysis for the MF category, we observed significant enrichment of candidate genes in five key terms: extracellular matrix structural constituent (GO:0005201), glycosaminoglycan binding (GO:0005539), heparin binding (GO:0008201), sulfur compound binding (GO:1901681), and organic acid binding (GO:0043177). KEGG pathway analysis further identified 23 significantly enriched pathways (*p* < 0.05) (Supplementary Table S2), including cytoskeleton in muscle cells (hsa04820), extracellular matrix (ECM)-receptor interaction (hsa04512), ABC transporters (hsa02010), malaria (hsa05144), and Staphylococcus aureus infection (hsa05150). Additionally, the PPI network revealed that 206 genes were involved in complex interaction relationships, such as EPBA2 and SLC4A1 (Figure 2D). These results highlighted immune response and ECM remodeling as central features of OS, offering potential entry points for therapeutic intervention.

### 2.2. Total of 11 Core Bioactive Compounds Were Determined

We retrieved 191 bioactive compounds corresponding to 38 substances from the official TCMSP database (accessed via https://www.tcmsp-e.com/tcmsp.php) (accessed on 17 July 2025), while 823 compounds from 16 additional related substances were obtained from the BATMAN-TCM database (available at http://bionet.ncpsb.org.cn/batman-tcm/) (accessed on 17 July 2025). A total of 983 unique compounds after deduplication, resulting in 1183 SMILES codes (Supplementary Table S3). GraphBAN-based prediction identified 628 candidate interactions between proteins encoded by DEGs and bioactive compounds, implicating 246 candidate target genes and 37 candidate bioactive compounds (Supplementary Table S4). Additionally, drug-likeness screening using Lipinski’s Rule of Five revealed that 27 of the 34 candidate compounds met ≥3 criteria (Table 1). Due to the limitation that SwissADME did not support overly long SMILES strings, only 34 bioactive compounds were included in the complete drug-likeness evaluation. Toxicity profiling (cardiac, hepatic, carcinogenic, ocular, and cytotoxic endpoints) further identified 11 core bioactive compounds with low-risk profiles (predicted probability < 0.5 for all toxicities) (Table 2). A total of 54 targets were associated with the core bioactive compounds and were defined as core target genes.

### 2.3. MFH-Derived Bioactive Compounds Demonstrated Multi-Target Regulatory Effects with Therapeutic Potential for OS

**Figure 3:**
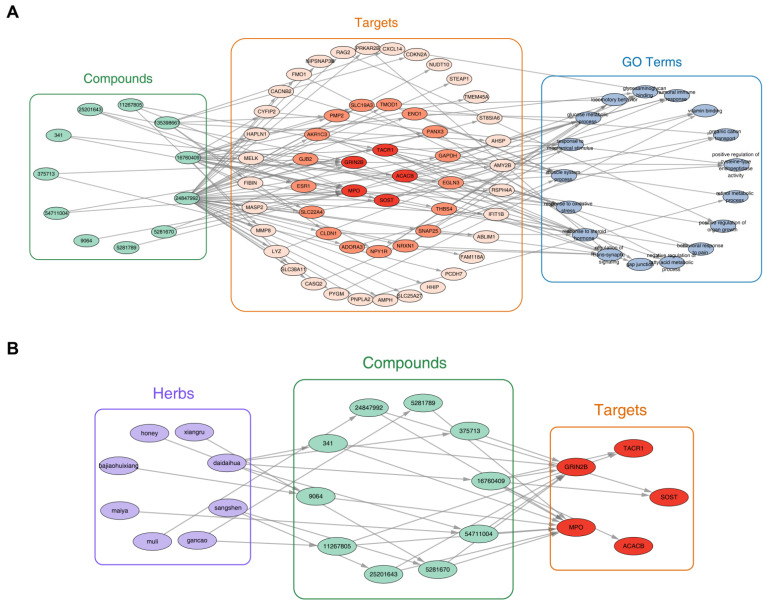
Candidate key active ingredients–key targets–pathway regulation network. (**A**) All compounds–targets–GO terms. (**B**) Highest connectivity of herbs–compounds–targets.

Through the core bioactive compound-target-pathway network, we found core target genes were collectively enriched in 17 GO terms, with most related to critical processes: regulation of trans-synaptic signaling, response to oxidative stress, response to steroid hormone, and response to mechanical stimulus (Figure 3A). Among these, five targets with the highest cumulative connectivity between core bioactive compounds and GO terms were selected, and their corresponding genes were identified as key target genes. These five key targets interacted with 10 key bioactive compounds (ellagic acid dihydrate (PubChem CID: 16760409), myricetin (1-) (PubChem CID: 25201643), digallic acid (PubChem CID: 341), 6,6′-dimethoxygossypol (PubChem CID: 375713), morin (PubChem CID: 5281670), digallate (PubChem CID: 54711004), (E)-3-[3,4-dihydroxy-5-(3-methylbut-2-enyl)phenyl]-1-(2,4-dihydroxyphenyl)prop-2-en-1-one (PubChem CID: 11267805), (+)-catechin (PubChem CID: 9064), Licoisoflavone (PubChem CID: 5281789), actonel with calcium (copackaged) (PubChem CID: 24847992) through 20 distinct interactions. Furthermore, the MFH substance-compound-target regulatory network integrated eight MFH substances: Daidaihua (Citrus aurantium), Gancao, Sangshen, Bajiaohuixiang (star anise), honey, Maiya, Muli, and Xiangru (Figure 3B). Within this network, three substances exhibited particularly high bioactivity potential. Daidaihua contributed four key bioactive compounds and Gancao and Sangshen each contributed two key bioactive compounds. Two key targets—MPO and GRIN2B—interacted with the majority of key bioactive compounds, suggesting their pivotal function in mediating multi-compound effects. Notably, several key bioactive compounds exhibited polypharmacological properties. Ellagic acid dihydrate (PubChem CID: 16760409) engaged all five key targets. Six compounds (e.g., morin (PubChem CID: 5281670)) each interacted with ≥two key targets. Digallate (PubChem CID: 54711004) and morin (PubChem CID: 5281670) originated from multiple MFH substances. These findings highlighted the polypharmacological nature of MFH-derived compounds and underscored their potential to modulate OS through multi-target interactions.

### 2.4. Molecular Docking Validated Strong Binding Affinities of Key Targets with MFH-Derived Compounds

**Figure 4:**
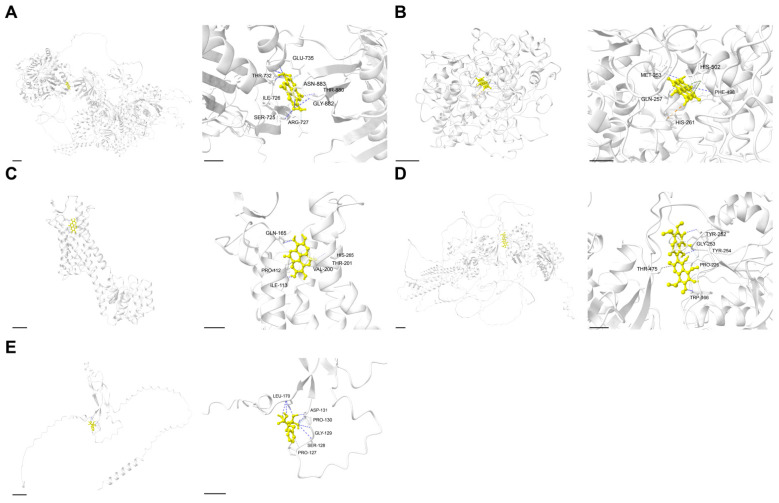
Molecular docking results of core active ingredients and core targets. (**A**) ACACB. (**B**) MPO. (**C**) TACR1. (**D**) GRIN2B. (**E**) SOST.Light gray: protein structure (receptor); bright yellow: active component (ligand).

We conducted molecular docking to verify the binding interactions between key targets and their corresponding bioactive compounds. All five tested complexes exhibited strong binding affinities, with binding energies ranging from −9.6 to −5.2 kcal/mol (Table 3). Ellagic acid dihydrate (PubChem CID: 16760409) demonstrated robust multi-target binding, interacting with ACACB, MPO, and TACR1. It achieved the strongest affinity with ACACB (−9.6 kcal/mol), stabilized by hydrogen bonds with residues such as THR-880 and ARG-727 (Figure 4A). Against MPO, it showed a comparable affinity (−9.2 kcal/mol), mediated by interactions with HIS-502 and GLN-257 (Figure 4B). For TACR1, it maintained strong binding (−8.4 kcal/mol) through residues including VAL-200 and GLN-165 (Figure 4C). In contrast, 6,6′-dimethoxygossypol (PubChem CID: 375713) bound stably to GRIN2B (−6.8 kcal/mol) via TYR-252 and TRP-166 (Figure 4D), while SOST interacted with actonel with calcium (copackaged) (PubChem CID: 24847992) (−5.2 kcal/mol) through LEU-170 and PRO-130 (Figure 4E). These findings supported the multi-target potential of MFH-derived compounds, particularly ellagic acid dihydrate, in modulating OS-related pathways.

### 2.5. MD Simulations Demonstrated Stable Complex Formation with Differential Interaction Forces

**Figure 5:**
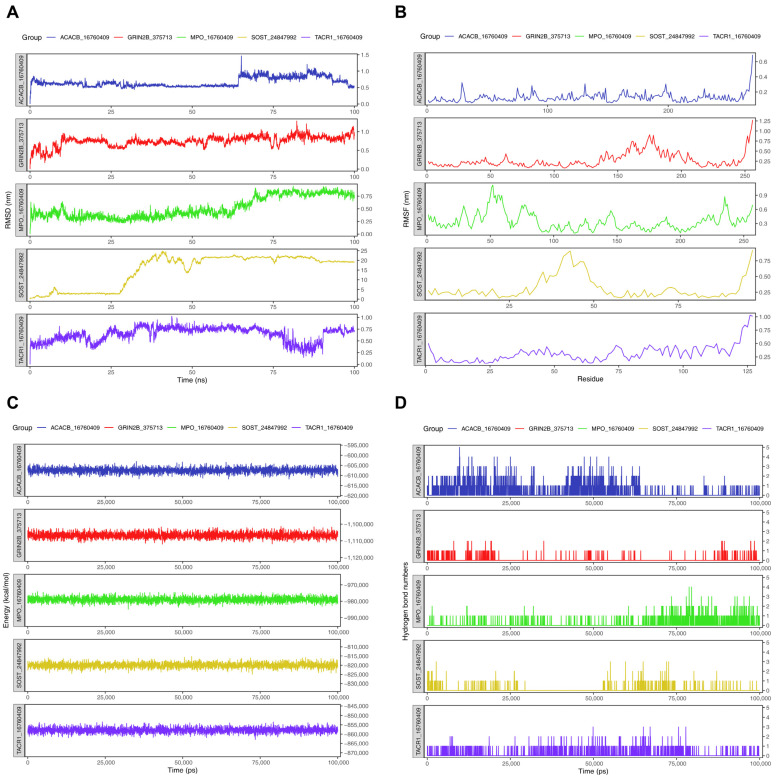
Molecular Dynamics (**A**) RMSD variation of molecular dynamics simulation. (**B**) RMSF variation in molecular dynamics simulation. (**C**) Energy changes in molecular dynamics simulations. (**D**) Hydrogen bond changes in molecular dynamics simulations.

We conducted molecular dynamics (MD) simulations to evaluate the structural stability and interaction dynamics of five key target-compound complexes. RMSD analysis demonstrated that four complexes—ACACB-ellagic acid dihydrate (PubChem CID: 16760409), MPO-ellagic acid dihydrate (PubChem CID: 16760409), TACR1-ellagic acid dihydrate (PubChem CID: 16760409), and GRIN2B-6,6′-dimethoxygossypol (PubChem CID: 375713)—maintained stable conformations throughout the 100 ns simulation, with RMSD values consistently below 25 nm (Figure 5A). In contrast, the SOST-actonel with calcium (copackaged) (PubChem CID: 24847992) complex exhibited higher structural fluctuations (pink trajectory), suggesting relative instability. Residue-specific flexibility was quantified by RMSF. All complexes showed similar fluctuation patterns, with heightened mobility primarily localized to C-terminal regions and intrinsically disordered loops (Figure 5B). This indicated that conformational flexibility was largely confined to expected structural domains rather than binding interfaces. Total energy trajectories revealed minimal deviations across all systems (Figure 5C). The absence of energy drift or abrupt transitions confirmed that each complex achieved thermodynamic equilibrium, supporting the physiological relevance of the docked conformations. Hydrogen bond analysis revealed distinct interaction patterns across the five complexes (Figure 5D). Overall, the number of hydrogen bonds remained within zero-two throughout most of the simulations. In ACACB-16760409 and MPO-16760409, transient increases to three–four hydrogen bonds were occasionally observed, suggesting that hydrogen bonding played a primary role in stabilizing these complexes, which might underlie their higher binding affinity and stability. By contrast, GRIN2B-375713, SOST-24847992, and TACR1-16760409 maintained negligible hydrogen bonding (<1 on average), indicating that their stability likely relied on alternative forces such as hydrophobic and electrostatic interactions. These differences in interaction profiles provided a mechanistic explanation for the differential binding stability.

### 2.6. Key Target Genes Function in Coordinated Networks Regulating Calcium Signaling and Lipid Metabolism in OS

**Figure 6:**
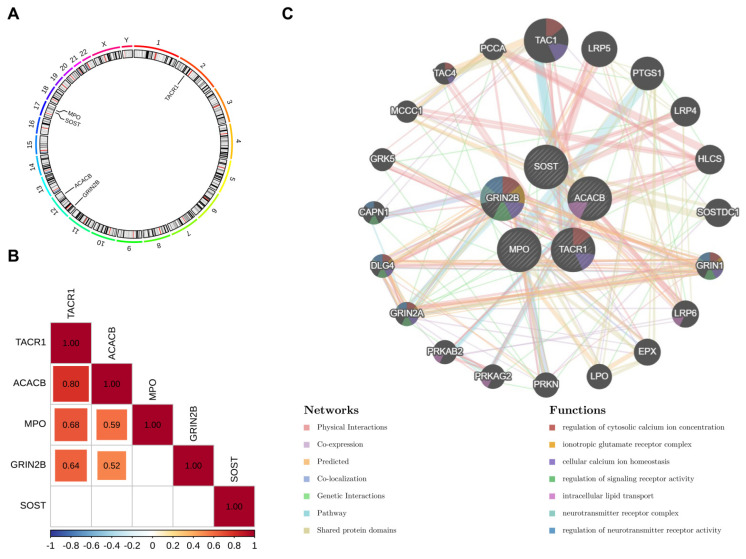
Chromosome analysis and GeneMANIA analysis (**A**) Chromosome location analysis of 5 core genes. (**B**) Correlation analysis of 5 core genes. (**C**) GeneMANIA analysis.

Chromosomal mapping, correlation, and functional network analyses further delineated the biological context of the key target genes. Chromosomal mapping revealed distinct genomic distributions of the five key target genes: TACR1 localized to chromosome 2, ACACB and GRIN2B to chromosome 12, and MPO and SOST to chromosome 17 (Figure 6A). This spatial segregation suggested potential co-regulation mechanisms within chromosomal domains. Correlation analysis demonstrated significant positive co-expression relationships (*p* < 0.05) among key target genes. TACR1 exhibited strong correlations with ACACB (cor = 0.80), MPO (cor = 0.68), and GRIN2B (cor = 0.64). ACACB correlated with MPO (cor = 0.59) and GRIN2B (cor = 0.52) (Figure 6B). These findings indicated potential functional synergy between TACR1, ACACB, MPO, and GRIN2B in OS pathogenesis. We performed functional network analysis through the GeneMANIA tool (https://genemania.org/) (accessed on 28 July 2025), which helped further illuminate interactions between key target genes and associated genes (Figure 6C). GRIN2B formed physical interactions and co-expression links with GRIN1 and DLG4. MPO interacted with EPX and PTGS1. TACR1 networked with TAC1 and GRK5. SOST engaged with LRP5 and LRP6. ACACB connected to HLCS and MCCC1. Functional enrichment analysis revealed pathway-specific roles. GRIN2B and TACR1 co-enriched in regulation of cytosolic calcium ion concentration and cellular calcium ion homeostasis. GRIN2B additionally governed neurotransmitter receptor activity and formed ionotropic glutamate receptor complexes. ACACB primarily mediated intracellular lipid transport. Together, these findings suggested that key target genes might act in coordinated networks influencing calcium signaling and lipid metabolism, thereby contributing to OS progression.

### 2.7. GSEA Revealed Shared Core Pathways and Target-Specific Specializations of Five Key Target Genes

**Figure 7:**
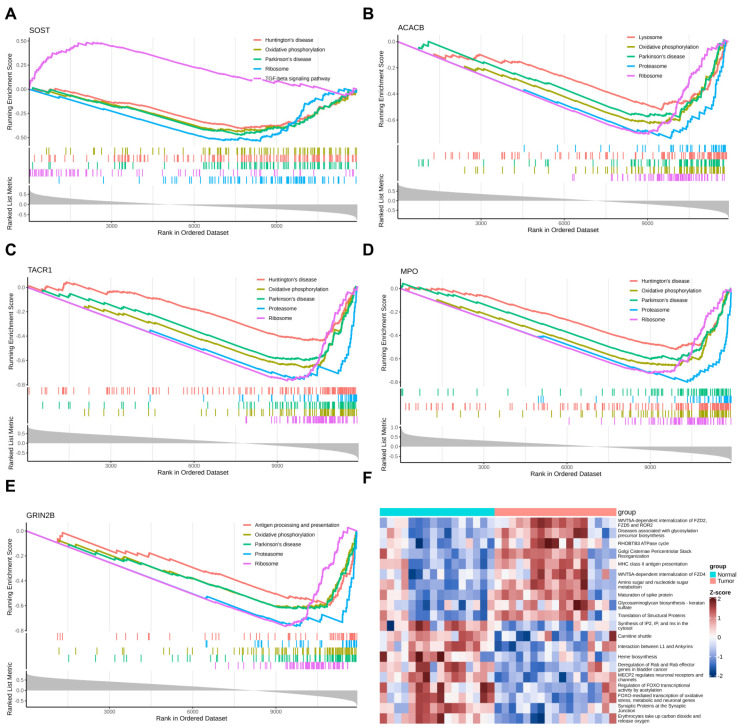
GESA and GSVA. (**A**) GSEA enrichment analysis of GRIN2B. (**B**) GSEA enrichment analysis of MPO. (**C**) GSEA enrichment analysis of ACACB. (**D**) GSEA enrichment analysis of TACR1. (**E**) GSEA enrichment analysis SOST. (**F**) GSVA.

GSEA revealed that all five key target genes—SOST, ACACB, TACR1, MPO, and GRIN2B—exhibited significant enrichment (*p*.adj < 0.05) in three core pathways, including ribosome, oxidative phosphorylation, and proteasome (Figure 7A–E and Supplementary Table S5–S9). This conserved enrichment pattern implicated these target genes in the coordinated control over the synthesis of proteins, energy metabolism, and the degradation of proteins. Notably, SOST (enriched in nine pathways) is uniquely associated with the TGF-beta signaling pathway, linking it to ECM remodeling. ACACB (44 pathways), TACR1 (35 pathways), and MPO (39 pathways) showed common enrichment for lysosome, highlighting roles in macromolecule catabolism. GRIN2B (36 pathways) was distinctively enriched in antigen processing and presentation, suggesting immune interface functions. These findings suggested that the five key target genes not only converged on essential cellular processes but also represented potential therapeutic entry points for pathway-specific intervention in OS.

### 2.8. GSVA Revealed Widespread Pathway Dysregulation Underlying Metabolic and Signaling Alterations in OS

GSVA revealed a total of 130 differential pathways between OS and normal groups, including 98 upregulated and 32 downregulated pathways in OS (*p*.adj < 0.05) (Figure 7F and Supplementary Table S10). In the OS group, activation was observed in the WNT signaling, glycometabolic reprogramming, and protein synthesis and modification (golgi cisternae pericentriolar stack reorganization; translation of structural proteins; maturation of spike protein). Conversely, several pathways were downregulated in OS, including those related to energy metabolism (carnitine shuttle; heme biosynthesis) and cellular signaling and homeostasis. These alterations might reflect underlying biological processes of metabolic dysregulation, inflammatory activation, and disruption of cellular homeostasis in OS.

### 2.9. Immune Infiltration Profiling Uncovered Altered Immune Cell Populations and Gene-Immune Correlations in OS

**Figure 8:**
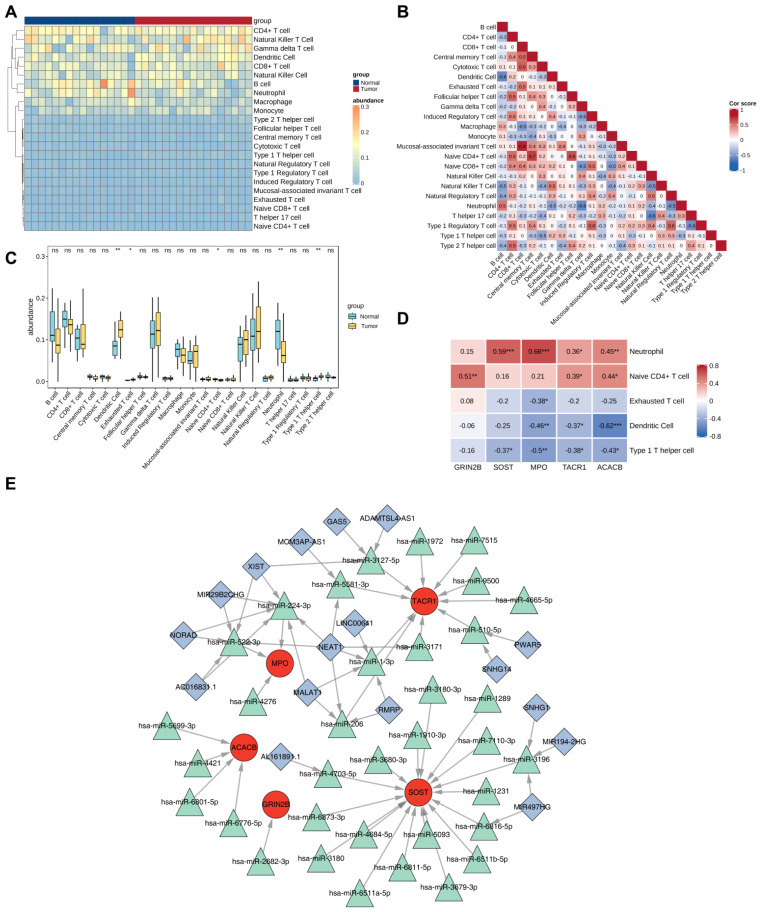
Immune infiltration profiling and lncRNA-miRNA-mRNA network (**A**) 23 kinds of immune cell abundance heat map. (**B**) 23 heat maps related to immune cells. (**C**) The abundance of 23 immune cells in the OS sample and the control sample. (**D**) Correlation heat map of 23 immune cells and 5 core targets. (**E**) lncRNA-miRNA-mRNA network (red: key target gene, blue: lncRNA, green: miRNA). Statistical significance: ns., not significant; * *p* < 0.05; ** *p* < 0.01; *** *p* < 0.001.

After excluding cell types undetectable in >50% of samples, 23 immune cell populations between OS and normal samples were analyzed to characterize immune cell infiltration patterns in OS. Among these, B cells, CD4+ T cells, gamma delta (γδ) T cells, dendritic cells (DCs), and natural killer T (NKT) cells exhibited consistently high abundance in both groups (Figure 8A). Spearman correlation analysis revealed significant intercellular relationships (|cor| > 0.3, *p* < 0.05). CD8+ T cells showed strong positive correlations with mucosal-associated invariant T cells (cor = 0.8), cytotoxic T cells (cor = 0.6), exhausted T cells (cor = 0.5), central memory T cells (cor = 0.4), and naive CD8+ T cells (cor = 0.4). Naive CD4 T cells correlated positively with central memory T cells (cor = 0.7), follicular helper T (Tfh) cells (cor = 0.6), and CD4+ T cells (cor = 0.5). Neutrophils demonstrated positive correlation with B cells (cor = 0.5) but negative correlations with dendritic cells (cor = −0.5), γδ T cells (cor = −0.6), natural regulatory T cells (nTregs) (cor = −0.5), and NKT cells (cor = −0.4) (Figure 8B). Comparative analysis identified five differentially infiltrated immune cells in OS (*p* < 0.05) (Figure 8C and Supplementary Table S11). Specifically, naive CD4 T cells and neutrophils were significantly decreased, while DCs, exhausted T cells, and type 1 T helper (Th1) cells increased. Correlation between key target genes and differentially infiltrated immune cells further revealed: GRIN2B positively correlated with naive CD4 T cells (cor = 0.5); SOST positively correlated with neutrophils (cor = 0.6) but negatively with Th1 cells (cor = −0.4); MPO positively correlated with neutrophils (cor = 0.7) but negatively with exhausted T cells (cor = −0.4), DCs (cor = −0.5), and Th1 cells (cor = −0.5); TACR1 and ACACB both showed positive correlations with neutrophils (TACR1: cor = 0.4; ACACB: cor = 0.5) and naive CD4 T cells (cor = 0.4), and negative correlations with DCs (TACR1: cor = −0.4; ACACB: cor = −0.6) and Th1 cells (cor = −0.4) (Figure 8D). Overall, these results indicated significant changes in the OS immune microenvironment that were observed. Negative correlation profiles between five key target genes and dysregulated immune cell subsets implied potential regulatory roles of these genes in immune evasion mechanisms.

### 2.10. The lncRNA-miRNA-mRNA Network Highlighted Uneven Post-Transcriptional Control of Five Key Target Genes

To elucidate the post-transcriptional regulation of the five key target genes (SOST, TACR1, ACACB, MPO, and GRIN2B), a comprehensive lncRNA-miRNA-mRNA network was constructed. Predictions from the miRDB (http://www.miRDB.org/ (accessed on 6 August 2025)) and TargetScan (https://www.targetscan.org/vert_80/ (accessed on 6 August 2025)) databases initially identified 113 and 213 miRNA-mRNA interactions, respectively, and their intersection yielded 35 high-confidence miRNA-mRNA pairs involving 35 unique miRNAs. Subsequently, ENCORI database (https://rnasysu.com/encori/ (accessed on 6 August 2025)) mining revealed 580 lncRNA-miRNA interactions, from which 31 validated pairs comprising 17 lncRNAs and 11 miRNAs were retained. The lncRNA-miRNA-mRNA network integrating these datasets illustrated multilayered regulation of the five key target genes: SOST was regulated by 17 miRNAs, TACR1 by 10, ACACB by four, MPO by three, and GRIN2B by a single miRNA (Figure 8E). Hub miRNAs exhibited extensive cross-talk, with hsa-miR-224-3p regulated by six lncRNAs, hsa-miR-522-3p by five, and hsa-miR-1-3p by four, while pivotal lncRNAs demonstrated broad regulatory capacity, notably NEAT1 targeting six miRNAs, XIST modulating three, and MALAT1 regulating three. Collectively, this network revealed intricate regulatory hierarchies, with NEAT1, XIST and malat1 emerging as important regulators through their control of multiple miRNAs targeting MPO and TACR1, while the disproportionate regulation of SOST (17 miRNAs) compared with GRIN2B (one miRNA) suggested differential post-transcriptional control of OS-associated genes.

### 2.11. Ellagic Acid Inhibits the Proliferation, Migration of 143B Cell

**Figure 9:**
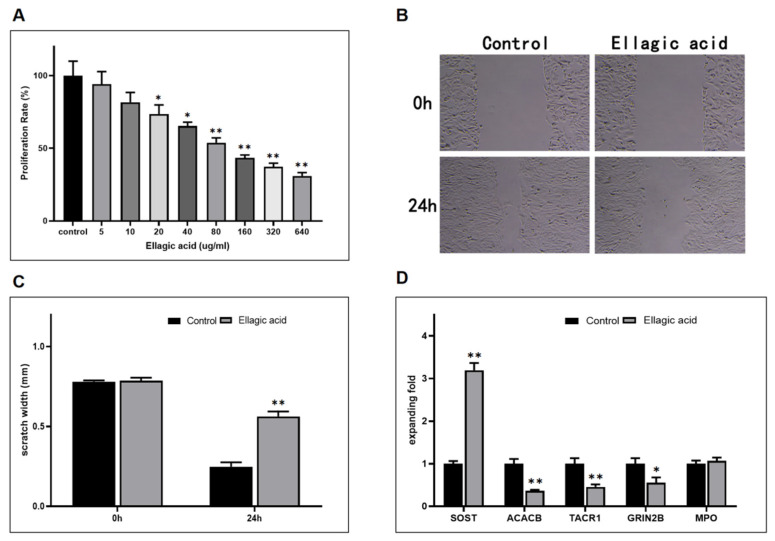
Effects of ellagic acid on 143B cell proliferation, migration, and core targets. (**A**) Effect of ellagic acid proliferation of 143B cell. (**B**) Scratch assay showing ellagic acid (40 μM) effects on 143B cell migration at 0 and 24 h, 50×; scale bar, 100 μm (*n* = 3). (**C**) Quantification of migration rate. (**D**) Quantification of core targets expression. Data are presented as mean ± SD. Statistical significance: * *p* < 0.05; ** *p* < 0.01.

Considering that ellagic acid dihydrate was identified as capable of concurrently targeting all five key genes, we proceeded to employ ellagic acid extract for an initial experimental validation of our findings. We measured the viability of 143B cells treated with ellagic acid extract. We found that cellular activity of 143B cells reduced with higher concentrations of ellagic acid (Figure 9A, Supplementary File S1). Afterwards, ellagic acid’s inhibitory role in 143B cell migration was confirmed (Figure 9B,C, Supplementary File S3).

### 2.12. Effect of Ellagic Acid on mRNA Expression of Core Target

We utilized qRT-PCR to validate the key analysis results of ellagic acid. 143B cells were treated with moderate concentrations of ellagic acid for 24 h, followed by mRNA expression level measurements of key target, including SOST, ACACB, TACR1,GRIN2B, and MPO.Compared with the Con group, the ellagic acid group (40 μg/mL) significantly decreased the mRNA expression levels of ACACB, TACR1, and GRIN2B, and increased that of SOST. However, there was no significant difference in MPO mRNA expression between the ellagic acid-treated group and the control group (Figure 9D, Supplementary File S2).

## 3. Discussion

OS is the most common primary malignant bone tumor in adolescents and young adults. Its high invasiveness and resistance to chemotherapy remain major challenges in clinical treatment [29,30]. Studies have shown that active components in flavonoids—such as quercetin and luteolin—can effectively inhibit the development and progression of osteosarcoma by interfering with key signaling pathways such as PI3K/Akt and Wnt/β-catenin [31,32]. Additionally, baicalin induces ferroptosis in osteosarcoma cells via the novel Nrf2/xCT/GPX4 regulatory axis, opening new avenues for developing safe and efficient natural antioxidative stress drugs [33]. By integrating network pharmacology, molecular simulation, and experimental validation, we for the first time systematically clarify the multi-target interaction profile of food-borne medicinal substances in intervening in osteosarcoma. It successfully identified 10 key active components, including ellagic acid dihydrate, and their corresponding 5 core targets (SOST, ACACB, TACR1, GRIN2B, MPO). These findings not only reveal the multi-component, multi-target, and multi-pathway synergistic characteristics of MFH substances but also provide an important theoretical basis and candidate molecules for developing low-toxicity natural therapies for OS.

In this study, we identified 317 DEGs between OS and normal bone samples and noted that these DEGs showed significant enrichment in pathways like humoral immune response, defense response to bacteria/fungi, and extracellular matrix (ECM) organization/ECM-receptor interaction (see Result 2.1), indicating that immune imbalance and ECM remodeling may represent key molecular features of OS. This finding aligns with current understanding of the OS tumor microenvironment (TME): components of the TME (immune cells, fibroblasts, ECM, etc.) drive OS progression and metastasis by regulating adhesion, migration, and invasion. Moreover, ECM remodeling and ECM-receptor interactions are closely associated with metastatic potential [34,35,36]. Simultaneously, an abnormal immune microenvironment has been linked to chemotherapy resistance and immunotherapy response. Recent studies have highlighted the critical roles of T cell lineages and dendritic cells (DCs) in OS, providing a rationale for combined or sequential immunotherapy strategies [37,38,39]. Based on this evidence, our enrichment results not only confirm the central role of immune and ECM pathways in OS at the transcriptomic level but also provide a clear starting point for subsequent intervention strategies targeting multiple pathways using active components derived from MFH substances. To preliminarily validate this multi-targeting strategy, we performed in vitro experiments which demonstrated that ellagic acid could inhibit the migration of 143B cells, reduce the mRNA expression levels of ACACB, TACR1, and GRIN2B, and elevate SOST expression, while showing no significant effect on MPO expression.

The five key targets identified in this study constitute a complex regulatory network involving bone metabolism, neural signaling, energy metabolism, and immune inflammation, revealing the potential dimensions of MFH substance intervention in osteosarcoma. The role of SOST (sclerostin) in osteosarcoma extends far beyond inhibiting osteogenic differentiation, as it is a classical antagonist of the WNT signaling pathway. Within the OS microenvironment, aberrant expression of SOST may disrupt the normal osteoblast-osteoclast balance by inhibiting osteoblast activity, thereby creating an environment conducive to tumor cell proliferation and invasion. More importantly, the WNT signaling pathway is closely associated with cancer stem cell properties. SOST may influence the self-renewal of osteosarcoma stem cells by modulating this pathway, thereby linking it to tumor recurrence and drug resistance [40,41,42]. The potential significance of MFH components intervening in SOST lies in their ability to potentially regulate bone metabolism bidirectionally and target cancer stem cells. ACACB is the rate-limiting enzyme in fatty acid synthesis [43]. By upregulating ACACB, OS cells accelerate fatty acid synthesis, which not only provides raw materials for the construction of rapidly proliferating cell membranes but also meets their energy demands under stressful conditions such as hypoxia through lipid energy storage. Furthermore, specific lipid signaling molecules themselves act as important proliferation signals. Therefore, targeting ACACB is a strategy to cut off the supply line for osteosarcoma cells at the metabolic source, potentially inducing metabolic stress and apoptosis. TACR1, which encodes the neurokinin-1 receptor (NK-1R), serves as the receptor for substance P and has been shown to promote tumor proliferation in various cancers including glioma and non-small cell lung cancer [44,45,46]. GRIN2B (GluN2B), a subunit of the N-methyl-D-aspartate receptor (NMDA receptor), is involved in the regulation of calcium signaling homeostasis. Its aberrant expression has been closely linked to invasiveness and metastasis in multiple solid tumors [47,48,49,50]. MPO (myeloperoxidase), primarily derived from neutrophils, plays a dual role in inflammation and immune regulation by influencing oxidative stress and shaping the tumor microenvironment through modulation of immune cell infiltration [51,52]. This evidence is consistent with the findings from our network analysis, indicating that these five key target genes may contribute to OS progression through multi-level pathways.

Based on these findings, this study further identified 10 key active components that interact with the aforementioned key target genes. Among these compounds, studies have shown that ellagic acid can simultaneously interfere with multiple processes like tumor cell proliferation, EMT/metastasis, inflammation, and immune escape, thereby exerting anti-tumor activity through pathways including PI3K/AKT, STAT3, and Wnt/β-catenin [53,54,55]. This study also confirmed its stable binding to ACACB, MPO, and TACR1. Digallate/Digallic Acid compounds have been widely reported in recent years to possess anti-tumor potential and are often combined with strategies such as drug carriers to improve pharmacokinetic properties [56,57]. Morin has been demonstrated to induce apoptosis and inhibit proliferation in osteosarcoma cells [58]. Myricetin exhibits broad-spectrum anti-cancer potential, acting through multiple signaling networks such as MAPK, EGFR, and TGF-β/Smad-EMT [59]. Chalcone and its derivatives have shown anti-proliferative, anti-angiogenic, and pro-apoptotic effects in various cancer studies, potentially related to the regulation of the JAK/STAT pathway [60,61,62]. (+)-Catechin, a natural polyphenol, has been confirmed to regulate oxidative stress and angiogenesis, thereby inhibiting tumor progression [63,64,65,66]. Licoisoflavone (e.g., Licoisoflavone A) induces G1/S arrest by inhibiting the CDK2-Cyclin E1 axis and effectively suppresses colorectal cancer growth in animal models [67]. Furthermore, 6,6′-dimethoxygossypol demonstrates stronger anti-tumor activity compared to its parent compound gossypol, supporting its potential as an anti-OS component [68,69]. Alendronate sodium, owing to its bone-seeking properties, can exert both anti-bone resorption and anti-tumor effects within the bone-related tumor microenvironment [70]. We found that these findings align with the results of molecular docking and molecular dynamics simulations, thereby highlighting their multi-target regulatory potential in OS progression.

Further investigation into the origin of the key active ingredients identified them as primarily derived from eight medicinal food homologous substances, including Daidaihua, Gancao, and Sangshen. Modern pharmacological research corroborates the individual anti-tumor or immunomodulatory potential of these substances [71,72,73,74,75,76,77,78,79,80,81,82]. For validating the reliability of network pharmacology predictions, we demonstrated that ellagic acid significantly inhibits the proliferation and migration of 143B osteosarcoma cells. The treatment downregulated the gene expression of core targets such as ACACB, TACR1, and GRIN2B, while upregulating the expression of SOST. The underlying mechanism may involve the inhibition of WNT signaling pathway, potentially leading to induced cell differentiation, modulated cell cycle progression, or impaired nuclear translocation of β-catenin, ultimately resulting in the suppression of malignant proliferation and migration in 143B cells. These findings not only mutually confirm the network-based predictions, providing preliminary evidence for the biological relevance of the computationally screened targets, but also highlight the potential application value of ellagic acid as a source for anti-osteosarcoma agents. Nevertheless, this in vitro study serves as preliminary validation; the actual distribution, metabolism, and synergistic effects of the active ingredients necessitate further investigation utilizing animal models and subsequent studies.

In this study, we found via GSEA that the five key target genes were jointly enriched in three core pathways: ribosome, oxidative phosphorylation, and proteasome. This suggests that the coordinated axis of protein synthesis—mitochondrial energy supply—protein degradation may serve as an important foundation for cellular homeostasis and adaptive survival in OS. Consistent with this, recent studies have shown that OS cells commonly exhibit activated oxidative phosphorylation programs and mitochondrial adaptive remodeling, which drive tumor growth and influence treatment response. Inhibition of the nuclear receptor RORγ can downregulate oxidative phosphorylation and suppress OS progression, indicating that the energy metabolism axis is a druggable target [83]. Meanwhile, abnormalities in the ubiquitin-proteasome system in OS have been systematically reviewed, involving multi-level regulation of tumor cell cycle, apoptosis, and metastasis. Related targets show translational potential, aligning with the observed enrichment direction of the “proteasome” pathway [84,85]. In terms of pathway specificity, our analysis revealed that the enrichment signals of SOST linked to TGF-β/ECM remodeling match recent knowledge of the bone tumor microenvironment’s biology: TGF-β drives matrix remodeling and immunosuppression, thus promoting OS invasion and migration. As a classical WNT inhibitor, SOST exhibits tumor-suppressive effects in OS models and has been proposed as a potential therapeutic target, suggesting that the WNT–bone matrix–TGF-β axis may collectively participate in shaping the OS microenvironment and driving progression [86,87]. Furthermore, at the GSVA level, we observed activation of WNT signaling, glycometabolic/glycosylation reprogramming, and upregulation of protein synthesis/modification, which echoes recent reviews and experimental studies: Wnt/β-catenin imbalance is a key driver of OS invasion, metastasis, and drug resistance; glycosylation abnormalities are associated with tumor immune escape and extracellular matrix remodeling; and molecular network alterations related to glycosylation/glycosaminoglycan pathways have also been identified in OS [88,89]. Overall, the dysregulation of both metabolism and signal transduction provides a multi-target intervention window for OS, and this matches the multi-pathway, multi-target regulatory characteristics found in MFH active components within this study.

Immunoinfiltration analysis in this study revealed that OS tissues exhibited a significant increase in the infiltration of DCs and exhausted T cells, coupled with a marked reduction in naive CD4^+^ T cells, compared to normal tissues. This distinct cellular profile delineates a dysfunctional tumor microenvironment (TME). The increased presence of DCs may suggest the initiation of antigen-presenting functions; however, the concurrent upregulation of T cell exhaustion markers and the loss of regenerative naive T cells collectively point to a state of ineffective anti-tumor immune response [90,91,92].

MPO represents the neutrophil/granulocyte oxidative stress axis, and its pro-inflammatory and immunomodulatory roles in tumorigenesis have been supported by numerous recent reviews and studies. MPO activity not only influences tumor redox homeostasis but also shapes the tumor microenvironment (TME), thereby affecting immune infiltration patterns—consistent with the observed MPO–neutrophil association. TACR1 (NK-1R) has been associated with tumor promotion in multiple cancer types, and direct evidence in OS demonstrates that NK-1R is overexpressed and could act as a therapeutic target, suggesting that the neuropeptide signaling axis may contribute to the observed immune and metabolic crosstalk [93]. The extract of bitter orange flower can downregulate the expression of TACR1, effectively cutting off that detrimental neural signaling, thereby potentially alleviating partial suppression of dendritic cells (DCs) and Th1 cells. This may contribute to the restoration of effective anti-tumor immunity [44]. The upregulation of MPO may induce an acute, high-intensity oxidative burst, directly triggering ferroptosis or apoptosis in tumor cells. The net effect shifts from “suppressing immune cells” to “directly killing tumor cells,” which aligns with the observed inhibition of cell proliferation and migration. This discovery significantly enriches our understanding of the multi-target and network-based mechanisms of action of MFH substances.

However, this study has several limitations. First, the screening based on public databases and computational simulations carries inherent risks of false positives and cannot fully capture the dynamic regulatory processes within biological systems. Second, while the cell-based validation was performed using the purified key compound (ellagic acid), the experimental scope remains limited. Dose-dependency, comprehensive cytotoxicity profiles, and safety assessments in normal cell lines were not fully explored. Furthermore, the conclusions drawn from a single public transcriptomic dataset require further validation with larger, independent cohorts to ensure generalizability. Future work should prioritize systematic in vitro and in vivo functional studies, detailed mechanistic investigations, and rigorous safety evaluations of the prioritized compound.

Overall, evidence from multiple levels—pathway enrichment, GSVA perturbation, immune infiltration, and key target genes—converges to support that OS progression is driven by the coupled effects of energy metabolism and protein homeostasis imbalance, WNT/ECM/TGF-β-mediated microenvironment remodeling, and an immunosuppressive network. Within this framework, multi-target MFH active components, such as ellagic acid dihydrate, possess the potential to concurrently modulate these key nodes.

## 4. Materials and Methods

### 4.1. Data Acquisition

Gene expression profiles were obtained from the GEO database (https://www.ncbi.nlm.nih.gov/geo/ (accessed on 17 July 2025)) by downloading the GSE99671 dataset (platform GPL20148). This dataset contained transcriptomic data derived from bone samples, originally comprising 18 OS samples and 18 normal samples. To ensure data quality, 15 formalin-fixed paraffin-embedded (FFPE) samples were excluded. Subsequently, outlier detection was performed using principal component analysis (PCA) and expression heatmap visualization. PCA was computed using the “DESeq2” package (v 1.40.2) [94], and visualized using the “ggplot2” package (v 3.5.2) [95]. Expression heatmap visualization was conducted using the “ComplexHeatmap” package (v 2.14.0) [96]. Based on the PCA and heatmap results, one OS sample and two normal samples were identified as outliers and excluded from further analysis. Thus, the final training cohort consisted of 16 OS samples and 17 normal samples.

### 4.2. Differential Expression Analysis

We conducted differential expression analysis on the GSE99671 dataset with the aim of identifying differentially expressed genes (DEGs) between OS patients and normal controls. We performed differential expression analysis on the GSE99671 dataset to identify differentially expressed genes (DEGs) between OS patients and normal controls. For this analysis, the “DESeq2” package (v 1.40.2) was used, and DEGs were filtered based on adjusted *p*-value (*p*.adj) < 0.05 and |log_2_FC| > 1. To visualize the distribution of DEGs, a volcano plot was generated using the “ggplot2” package (v 3.5.2). The top 10 upregulated and top 10 downregulated genes, ordered by log_2_FC values in descending order, were marked on the volcano plot. Additionally, a heatmap illustrating the expression patterns of these 20 top DEGs (10 upregulated and 10 downregulated) was created using the “ComplexHeatmap” package (v 2.14.0).

### 4.3. Functional Enrichment and Protein–Protein Interaction (PPI) Analysis

Functional enrichment analysis was performed on the identified DEGs to elucidate their biological significance. GO, BP, CC, and MF categories, and KEGG pathway analysis were conducted using the “clusterProfiler” package (v 4.8.1) [97] (*p* < 0.05). To explore functional interactions among candidate genes, we constructed PPI networks using the online Search Tool for the Retrieval of Interacting Genes (STRING) database (https://string-db.org (accessed on 20 July 2025)). Interactions with a confidence score > 0.7 were retained. Genes exhibiting fewer than two interactions with other genes were excluded from the network. Finally, the retained PPI relationships were visualized using the “circlize” package (v 0.4.16) [98].

### 4.4. Identification of Bioactive Compounds from 106 Medicine–Food Homologous (MFH) Substances

Bioactive compounds from 106 MFH substances were systematically collected using a dual-database approach. We first conducted a query in the Traditional Chinese Medicine Systems Pharmacology Database and Analysis Platform (TCMSP, https://www.tcmsp-e.com/tcmsp.php (accessed on 17 July 2025)) to collect the primary chemical constituents of the target substance. Bioactive compounds that satisfied the screening criteria—specifically oral bioavailability (OB) ≥ 30% and drug-likeness (DL) ≥ 0.18—were further retained. For chemical substances that were not cataloged in the TCMSP database, we carried out supplementary searches via the Bioinformatics Analysis Tool for Molecular Mechanism of Traditional Chinese Medicine (BATMAN-TCM, http://bionet.ncpsb.org.cn/batman-tcm/ (accessed on 17 July 2025)). Data from two databases were merged, and duplicate entries were removed to generate a nonredundant compound list. Subsequently, we obtained the SMILES representations of these bioactive compounds from the PubChem database (https://pubchem.ncbi.nlm.nih.gov/ (accessed on 17 July 2025)) to facilitate follow-up computational analyses. Notably, some bioactive compounds were shared by multiple MFH substances, and in certain cases, a single SMILES structure corresponded to multiple PubChem Compound Identifiers (CIDs). In addition, stereochemical information of chiral atoms was not explicitly represented in the retrieved SMILES strings.

### 4.5. Prediction of Candidate Interactions

In this study, target genes referred to those genes among the DEGs that were predicted to interact with bioactive compounds, whereas targets denoted the protein products encoded by these genes, serving as the functional binding partners of bioactive compounds. GraphBAN, a graph neural network model integrating bidirectional attention mechanisms, was used to predict protein-compound interactions [99]. To identify candidate bioactive compounds with therapeutic potential for OS, protein-compound interactions were predicted using GraphBAN. Amino acid sequences of proteins encoded by DEGs were retrieved from the Universal Protein Resource (UniProt) database (https://www.uniprot.org/ (accessed on 20 July 2025)) and saved in FASTA format. Three pre-trained sub-models within GraphBAN—BioSnap, KIBA, and BindingDB—were used to independently predict interaction probabilities for each protein–compound pair. Only pairs with predicted probabilities > 0.5 in all three sub-models were retained as candidate interaction pairs, and the compounds involved in these interactions were defined as candidate bioactive compounds.

### 4.6. Drug-likeness and Toxicity Evaluation

Candidate bioactive compounds were further evaluated for drug-likeness and toxicity. SMILES representations of these compounds were submitted to the SwissADME platform (http://www.swissadme.ch/ (accessed on 20 July 2025)) to predict physicochemical properties and drug-likeness according to Lipinski’s Rule of Five: (1) molecular weight ≤ 500 Da; (2) Moriguchi octanol–water partition coefficient (MLOGP) ≤ 4.15; (3) ≤10 hydrogen bond acceptors; (4) ≤5 hydrogen bond donors; and (5) topological polar surface area (TPSA) ≤ 140 Å2. Compounds meeting at least three of these criteria were retained for subsequent toxicity prediction using ADMETlab 2.0 (https://admetmesh.scbdd.com/ (accessed on 20 July 2025)) and ProTox3 (https://tox.charite.de/protox3/ (accessed on 20 July 2025)). Five toxicity endpoints were assessed: cardiotoxicity, hepatotoxicity, carcinogenicity, eye corrosivity, and cytotoxicity. Compounds exhibiting no high-risk signals in all endpoints (predicted probability < 0.5) were defined as core bioactive compounds, and the genes encoding their interacting proteins were designated as core target genes.

### 4.7. Identification of Key Target Genes and Bioactive Compounds

An integrated regulatory network was constructed to explore the associations among core bioactive compounds, core target genes, and GO terms. We visualized the target network (e.g., PPI network or herbal component-target network) using Cytoscape software (version 3.10.3) [100]. Genes encoding targets that exhibited the highest degree centrality were designated as key target genes, and the corresponding interacting core bioactive compounds were designated as key bioactive compounds. Furthermore, to delineate the relationships linking MFH substances, their derived key bioactive compounds, and the key target genes, an additional MFH substance-compound-target network was also generated in Cytoscape software (v 3.10.3).

### 4.8. Molecular Docking

To verify the relationship between key bioactive compounds and key target genes—specifically to assess their binding affinity—we conducted molecular docking via the CB-Dock2 software (https://cadd.labshare.cn/cb-dock2/ (accessed on 6 August 2025)), an online tool for protein-ligand docking analysis, using a blind docking approach without predefining binding sites. Initially, we retrieved the 3D structures of key bioactive compounds from ChemSpider (https://www.chemspider.com/ (accessed on 6 August 2025)), which were required for subsequent structural optimization in molecular docking. Meanwhile, the structures of proteins encoded by key target genes (key targets) were acquired from the RCSB Protein Data Bank (PDB) (https://www.rcsb.org/ (accessed on 6 August 2025)) by searching with the corresponding gene IDs of key targets. Based on the interaction probabilities predicted by the three GraphBAN sub-models (BioSnap, KIBA, BindingDB), one type of interaction with the highest average interaction probability for each key target was selected for CD-Dock2 molecular docking. In the molecular docking analysis of this study, we considered a binding energy of ≤−5 kcal/mol to signify good binding affinity between key bioactive compounds and their corresponding target proteins. Subsequently, the docking conformation PDB files generated by CB-Dock were visualized using ChimeraX (v 1.10) [101].

### 4.9. Molecular Dynamics (MD) Simulation

To validate the binding stability between key bioactive compounds and their corresponding key targets predicted by molecular docking, MD simulations were performed using GROningen MAchine for Chemical Simulations (GROMACS) (v 2024.4) [102]. The entire simulation process adhered to the rules of the AMBER99SB-ILDN force field, with the Three-Point Transferable Intermolecular Potential (TIP3) water model employed, and the simulation box was set to a cubic shape. We first set a 1-nanometer distance between the edge of the box and the protein edge to ensure sufficient space for solvent molecules in the molecular dynamics system. To maintain the electroneutrality of the entire system, counterions were added to offset any net charge from the protein-ligand complex. Next, we carried out energy minimization using the steepest descent method to reduce high-energy interactions within the system. After minimization, the system was processed through a canonical ensemble (NVT, where the number of particles, volume, and temperature are kept constant to reach thermal balance) and then an isothermal-isobaric ensemble (NPT, where the number of particles, pressure, and temperature are regulated to match physiological pressure conditions). During the NVT and NPT processes, the V-rescale method was used for temperature coupling, with a reference temperature set to 300 K, a time step of 2 femtoseconds, and the entire process lasting 100 picoseconds. The molecular dynamics simulation was set to run for 20 nanoseconds. The RMSD of the key bioactive compound-key gene-encoded protein complex, RMSF) of the protein, total energy, and number of hydrogen bonds were calculated.

### 4.10. Chromosomal Mapping, Correlation, and Functional Network Analyses of Key Target Genes

To further characterize the biological context of the key target genes, chromosomal mapping, correlation, and network analyses were performed. The “circlize” package (v 0.4.16) was used to visualize the chromosomal positions of key target genes. To assess co-expression patterns among key target genes, Spearman correlation analysis was performed using the “psych” package (v 2.5.3) based on the GSE99671 dataset [103]. Statistically significant correlations (|correlation coefficient (cor)| > 0.3, *p* < 0.05) were visualized as a heatmap using the “corrplot” package (v 0.95) [104]. Furthermore, key target genes were submitted to the Gene Multiple Association Network Integration Algorithm (GeneMANIA) database (https://genemania.org/) (accessed on 28 July 2025) to elucidate functional associations and biological pathways involving key target genes.

### 4.11. Gene Set Enrichment Analysis (GSEA) and Gene Set Variation Analysis (GSVA)

To investigate the functional enrichment patterns associated with the key target genes, GSEA was performed. We first retrieved the c2.cp.kegg legacy.v2025.1.Hs.symbols.gmt gene set—an ensemble of genes associated with KEGG pathways—from the Molecular Signatures Database (MSigDB, https://www.gsea-msigdb.org/gsea/msigdb (accessed on 6 August 2025)), and used it as the reference gene set to support subsequent GSEA enrichment analysis of OS differential genes. For each key target gene, its correlation with all other genes across samples in the GSE99671 cohort was computed using the “psych” package (v 2.5.3). This generated a list of genes ranked by descending correlation coefficient. Subsequently, we performed GSEA based on the expression matrix of OS differential genes using the “clusterProfiler” package (version 4.8.1), with the screening criteria set as adjusted *p*-value (*p*.adj) < 0.05 and absolute value of normalized enrichment score (|NES|) > 1.

To characterize biological pathway activity differences between OS and normal samples, GSVA was employed. We used the c2.cp.v2025.1.Hs.entrez.gmt gene set from MSigDB (https://www.gsea-msigdb.org/gsea/msigdb)—a gene set focused on canonical pathways—as the reference for GSVA. Subsequently, we calculated GSVA scores for all samples in the GSE99671 training set using the “GSVA” package (v 1.48.2) [105]. Differential GSVA scores between the OS and normal groups were then assessed using the “limma” package (v 3.56.2) (*p*.adj < 0.05) [106]. A heatmap was generated using the “ComplexHeatmap” package (v 2.14.0). Pathways with t > 0 were considered activated in the OS group, while pathways with t < 0 were considered activated in the normal group.

### 4.12. Immune Infiltration Analysis

To comprehensively compare immune cell abundance differences between OS and normal samples and identify immune cell populations potentially critical in OS pathogenesis, an immune infiltration analysis was performed. Gene expression data from the GSE99671 cohort were analyzed using the Immune Cell Abundance Identifier (ImmuCellAI) algorithm (https://guolab.wchscu.cn/ImmuCellAI/ (accessed on 6 August 2025)) to estimate the relative abundances of 24 predefined immune cell types [107,108]. Immune cell types that were not detected in >50% of the samples were excluded, resulting in 23 immune cell types retained for subsequent analyses. The abundance matrix of these 23 immune cell types across all samples was visualized as a heatmap using the “ComplexHeatmap” package (v 2.14.0). To investigate the interrelationships among immune cell populations, Spearman correlation analysis was conducted on the estimated abundances of the 23 immune cell types using the “psych” package (v 2.5.3). To clearly display the strength and direction of correlations within the resulting matrix (e.g., between GSVA pathway scores or key gene expressions), we visualized this correlation matrix as a heatmap using the “ComplexHeatmap” package (version 2.14.0) in R. To identify immune cell types significantly associated with OS pathogenesis, Wilcoxon tests were performed to compare the estimated abundances of each of the 23 immune cell types between OS and normal groups. Immune cell types exhibiting significantly different abundances (*p* < 0.05) were defined as differentially infiltrated immune cells, and the distribution for these cells was visualized using a boxplot generated with the “ggplot2” package (v 3.5.2). To explore potential regulatory relationships, Spearman correlation analysis was performed between key target genes and differentially infiltrated immune cells. Using the “psych” package (v 2.5.3), we performed this analysis to assess correlations between target variables (such as OS-related DEG expression and pathway activity scores). For correlations meeting the statistical significance criteria (|cor| > 0.3, *p* < 0.05), we further visualized them as a heatmap via the “ComplexHeatmap” package (v 2.14.0), which helps distinguish strong from weak correlation signals.

### 4.13. Construction of Long Non-Coding RNA (lncRNA)-microRNA (miRNA)-mRNA Regulatory Network

To elucidate the post-transcriptional regulatory mechanisms involving the key target genes identified, a comprehensive regulatory network was constructed. miRNAs targeting these key target genes were predicted using two databases, including MicroRNA DataBase (miRDB, http://www.miRDB.org/ (accessed on 6 August 2025)) and TargetScan (https://www.targetscan.org/vert_80/ (accessed on 6 August 2025)). Predicted miRNA-mRNA interactions were retained only if they met the following stringent criteria: miRDB Target Score > 75 and TargetScan context++ score < −0.25. Furthermore, only miRNAs predicted to target a key target gene by both databases were defined as targeting miRNAs. Subsequently, we predicted lncRNAs that potentially regulate these targeting miRNAs using The Encyclopedia of RNA Interactomes (ENCORI) database (https://rnasysu.com/encori/ (accessed on 6 August 2025))—a well-recognized resource specializing in RNA-RNA interaction predictions. Interactions were retained if they satisfied: clipExpNum > 5 (indicating experimental support from ≥ 5 CLIP-seq datasets) and pancancerNum > 0 (indicating pan-cancer evidence). Finally, we visualized the integrated lncRNA-miRNA-mRNA regulatory network—constructed by integrating previously predicted lncRNA-miRNA and miRNA-mRNA interaction relationships—using Cytoscape software (v 3.10.3).

### 4.14. Materials and Culture of 143B Cells

Ellagic acid was purchased from MedChemEXpress (Monmouth Junction, NJ, USA). Preparation Method: A volume of 10 mL of complete cell culture medium was used to dissolve 169.11 mg of the stock solution. The resulting mixture was then filtered through a 0.22 μm pore-size filter, followed by the addition of 40 mL of complete cell culture medium to achieve a final volume of 50 mL. The final concentration of the solution was adjusted to 10 μM/L. The 143B cells used in this study were preserved in our research group’s laboratory (Jiamusi, China) and cultured in F12K medium (BasalMedia, Shanghai, China). This medium was supplemented with 10% fetal bovine serum (FBS, GeminiBio, West Sacramento, CA, USA), as well as 100 U/mL penicillin and 100 μg/mL streptomycin (HyClone™, Logan, UT, USA). Cells were cultured at 37 °C in a humidified incubator containing 5% CO_2_, a condition that mimics the physiological environment to support optimal cell growth. For cell proliferation assays, 143B cells were seeded into 96-well plates at a density of 6 × 10^3^ cells per well and left to adhere to the plate surface for 24 h before subsequent treatments. Ellagic acid (0 to 640 μM) was applied for 2 h. After the treatment period, we added 10 μL of Cell Counting Kit-8 (CCK-8, Meilunbio, Dalian, China, #MA0218-L-JU1-29F) reagent to each well. The plates were then incubated for 2 h at 37 °C to allow sufficient color development, after which absorbance at 450 nm was measured using a microplate reader to quantify cell viability.

### 4.15. Wound Healing Assays

For the wound healing assay, we first prepared a 95% confluent monolayer of 143B cells and created a linear scratch using a sterile pipette tip. After applying the experimental treatments, the cells were incubated under standard conditions (37 °C, 5% CO_2_), and wound closure was monitored at 0 and 24 h using an inverted microscope, with images captured to quantify healing progress.

### 4.16. Quantitative Real-Time Polymerase Chain Reaction (qRT-PCR) Assay

We extracted total RNA from the cells using Trizol reagent (Servicebio, Wuhan, China), and assessed RNA purity via the OD260/280 ratio (targeting 1.8–2.0) to ensure sample integrity. Subsequently, the qualified RNA was reverse-transcribed into cDNA using the RevertAid First Strand cDNA Synthesis Kit (Thermo, Waltham, MA, USA), following the manufacturer’s protocol for primer annealing and extension. To quantify the relative expression levels of target genes, we conducted quantitative real-time PCR (qRT-PCR) using a Bio-Rad CFX Duet Fluorescent Quantitative PCR System. The reaction was performed with HieffTM qPCR SYBR^®^ Green Master Mix (No Rox Plus, catalog No. 11201ES) from Yeasen (Shanghai, China), following the manufacturer’s recommended protocol. The qPCR thermal profile started with a preliminary denaturation phase at 95 °C for 15 min to trigger DNA polymerase activity, followed by 40 rounds of amplification. Each round included denaturation at 95 °C for 10 s, annealing at 59 °C for 20 s, and extension at 72 °C for 30 s. Relative expression levels of target genes were computed using the 2^−ΔΔCt^ method, with GAPDH mRNA serving as the endogenous control to standardize against inter-sample differences. Comprehensive primer sequences are provided in Table 4.

### 4.17. Statistical Analysis

We implemented all bioinformatics analyses involved in this study using R software (version 4.3.3). We used the Wilcoxon test to assess differences in continuous variables between different groups. Statistical significance was set at *p* < 0.05 for all analyses.

## 5. Conclusions

This study systematically integrated multi-level approaches—including transcriptomic analysis, prediction of MFH-derived components, regulatory network construction, molecular docking, molecular dynamics simulations, GSEA, GSVA, immune infiltration analysis, post-transcriptional regulatory network analysis, and in vitro cell experiments—to successfully identify five key target genes (SOST, ACACB, TACR1, GRIN2B, MPO) and ten key active components (such as ellagic acid dihydrate, morin, and licoisoflavone) associated with OS. Furthermore, eight source MFH substances (including Citrus aurantium, Glycyrrhiza uralensis, and Morus alba) were traced. The results reveal the multi-target and multi-pathway regulatory characteristics of MFH substances in OS, encompassing integrated effects on protein homeostasis, energy metabolism, cellular signaling pathways, and the immune microenvironment, with particular emphasis on the multi-target binding potential of components such as ellagic acid dihydrate. This study provides systematic evidence clarifying the molecular mechanisms of MFH substances in OS prevention and treatment and establishes a theoretical foundation for developing safe and efficient natural anti-OS therapeutic strategies. However, this study has several limitations. First, the analysis primarily relied on public transcriptomic data and bioinformatic predictions, with a limited sample size and lack of validation in independent clinical cohorts, which may affect the generalizability of the findings. Second, the pharmacokinetic profiles, bioavailability, and in vivo stability of the identified MFH active components require further experimental validation. Third, although molecular docking and dynamics simulations serve as predictive tools, they cannot fully replace wet-lab validation; their results need to be confirmed through clinical studies and animal experiments. Finally, MFH substances exhibit complex compositions and diverse sources, and the synergistic effects among single versus multiple components remain unclear, posing challenges for practical clinical application.

In conclusion, this study provides new molecular insights into the application of MFH substances in OS, yet further validation through multi-omics approaches, in vivo experiments, and clinical research is essential to confirm their anti-osteosarcoma potential and safety.

## Figures and Tables

**Table 1:** Drug similarity assessment results of candidate active ingredients.

Molecule	MW	MLOGP	X.H.bond.acceptors	X.H.bond.donors	TPSA
145857	306.7	−0.18	5	0	87.41
24847992	383.2	−3.32	11	5	230.99
5281855	302.19	0.14	8	4	141.34
16760409	338.22	−1.4	10	6	159.8
890	660.04	−7.36	24	12	459.42
135398667	412.19	−2.95	12	5	225.94
21252321	683.7	−6.13	16	11	295.29
44145386	580.58	−1.34	13	5	174.99
65064	458.37	−0.44	11	8	197.37
5280343	302.24	−0.56	7	5	131.36
5281672	318.24	−1.08	8	6	151.59
5281680	318.24	−1.08	8	6	151.59
14515885	318.24	−1.08	8	6	151.59
25201643	317.23	−1.08	8	5	154.42
445642	808.01	−0.05	13	4	198.59
341	322.22	0.02	9	6	164.75
375713	546.61	2.43	8	4	133.52
392443	354.35	1.28	6	3	96.22
440735	288.25	0.16	6	4	107.22
5281654	316.26	−0.31	7	4	120.36
5281670	302.24	−0.56	7	5	131.36
54711004	321.22	0.02	9	5	167.58
11267805	340.37	2.14	5	4	97.99
9064	290.27	0.24	6	5	110.38
31161	316.26	−0.31	7	4	120.36
128853	303.24	−0.22	7	6	134.52
128861	287.24	0.32	6	5	114.29
392442	354.35	1.28	6	3	96.22
5280445	286.24	−0.03	6	4	111.13
5281789	354.35	1.09	6	4	111.13
11968812	286.24	−0.03	6	4	111.13
102004869	887.02	−2.35	18	10	276.14
5280965	887.02	−2.35	18	10	276.14
439533	304.25	−0.64	7	5	127.45

**Table 2:** Toxicity assessment results of candidate active ingredients.

PubChem CID	hERG_ADMElab	H.HT_ADMElab	Carcinogenicity_ADMElab	EC_ADMElab	Hepatotoxicity_ProTox	Cardiotoxicity_ProTox	Carcinogenicity_ProTox	Cytotoxicity_ProTox
145857	0.1	0.036	0.786	0.058	Inactive	Inactive	Inactive	Inactive
24847992	0.03	0.18	0.026	0.215	Inactive	Inactive	Inactive	Inactive
5281855	0	0.144	0.314	0.009	Inactive	Inactive	Active	Inactive
16760409	0	0.144	0.314	0.009	Inactive	Inactive	Inactive	Inactive
135398667	0.014	0.227	0.364	0.003	Inactive	Inactive	Inactive	Inactive
439533	0.07	0.176	0.039	0.003	Inactive	Inactive	Active	Inactive
5280343	0.099	0.1	0.05	0.007	Inactive	Inactive	Active	Inactive
5281672	0.145	0.099	0.028	0.008	Inactive	Inactive	Active	Inactive
5281680	0.117	0.112	0.05	0.004	Inactive	Inactive	Active	Inactive
14515885	0.124	0.13	0.085	0.003	Inactive	Inactive	Active	Inactive
25201643	0.145	0.099	0.028	0.008	Inactive	Inactive	Inactive	Inactive
341	0.015	0.407	0.018	0.058	Inactive	Inactive	Inactive	Inactive
375713	0.002	0.004	0.019	0.003	Inactive	Inactive	Inactive	Inactive
392443	0.063	0.229	0.689	0.003	Inactive	Inactive	Inactive	Inactive
440735	0.045	0.112	0.395	0.005	Inactive	Inactive	Active	Inactive
5281654	0.061	0.064	0.047	0.007	Inactive	Active	Inactive	Inactive
5281670	0.157	0.067	0.035	0.009	Inactive	Inactive	Inactive	Inactive
54711004	0.015	0.407	0.018	0.058	Inactive	Inactive	Inactive	Inactive
11267805	0.044	0.38	0.431	0.004	Inactive	Inactive	Inactive	Inactive
9064	0.022	0.071	0.09	0.003	Inactive	Inactive	Inactive	Inactive
31161	0.09	0.058	0.092	0.004	Inactive	Inactive	Active	Inactive
128853	0.153	0.193	0.04	0.007	Inactive	Inactive	Active	Inactive
128861	0.085	0.165	0.069	0.008	Inactive	Inactive	Active	Inactive
392442	0.059	0.233	0.554	0.003	Inactive	Inactive	Inactive	Inactive
5280445	0.064	0.084	0.095	0.009	Inactive	Inactive	Active	Inactive
5281789	0.027	0.298	0.091	0.004	Inactive	Inactive	Inactive	Inactive
11968812	0.015	0.152	0.043	0.003	Inactive	Inactive	Inactive	Active

**Table 3:** The docking binding energy between the core active ingredient and the core target molecule.

Gene_Name	PubChem CID	DockScore (kcal/mol)
ACACB	16760409	−9.6
GRIN2B	375713	−6.8
MPO	16760409	−9.2
SOST	24847992	−5.2
TACR1	16760409	−8.4

**Table 4:** Primers used in qRT-PCR.

Gene	Forward Primers (From 5′ to 3′)	Reverse Primers (From 5′ to 3′)
ACACB	AAGCGGCAGGAATAAAGTGATTT	ACTCTTGGTGATCGGCTTGG
GRIN2B	CAGGGTGTGCGAGGAAATCT	AGGATATGCATTCGGACGCC
MPO	GATGTGCAACAACAGACGCA	GAAGCCGTCCTCATACTCCG
SOST	CACACAGCCTTCCGTGTAGT	ACTCGGACACGTCTTTGGTC
TACR1	GCTGCCCTTCCACATCTTCT	CCCAGACGGAACCTGTCATT
GAPDH	GGAAGCTTGTCATCAATGGAAATC	TGATGACCCTTTTGGCTCCC

## Data Availability

Gene expression profiles are available in the Gene Expression Omnibus (GEO) database under accession GSE99671 (https://www.ncbi.nlm.nih.gov/geo/) (accessed on 17 July 2025). Bioactive compounds from medicine–food homologous (MFH) substances were from TCMSP (https://www.tcmsp-e.com/tcmsp.php) (accessed on 17 July 2025) and BATMAN-TCM (http://bionet.ncpsb.org.cn/batman-tcm/) (accessed on 17 July 2025). SMILES representations, target protein amino acid sequences, and 3D structures were retrieved from PubChem (https://pubchem.ncbi.nlm.nih.gov/) (accessed on 17 July 2025), UniProt (https://www.uniprot.org/) (accessed on 20 July 2025), and RCSB PDB (https://www.rcsb.org/) (accessed on 6 August 2025), respectively. miRNAs targeting key genes were predicted via miRDB (http://www.miRDB.org/) (accessed on 6 August 2025) and TargetScan (https://www.targetscan.org/vert_80/) (accessed on 6 August 2025), and lncRNA-miRNA interactions from ENCORI (https://rnasysu.com/encori/) (accessed on 6 August 2025). Other relevant data are available from the corresponding author upon reasonable request.

## Supplementary Materials

The following supporting information can be downloaded at: https://www.mdpi.com/article/10.3390/ijms27031360/s1.

## Abbreviations

The following abbreviations are used in this manuscript:

OS	Osteosarcoma
MFH	Medicine–food homologous
DEGs	Differentially expressed genes
qRT-PCR	Quantitative real-time polymerase chain reaction
GSEA	Gene set enrichment analysis
GSVA	Gene set variation analysis
PPI	Protein–protein interaction
GPX4	Glutathione peroxidase 4
Nrf2	Nuclear factor erythroid 2-related factor 2
NMDA	N-methyl-D-aspartate
DCs	Dendritic cells
Th1 cells	Type 1 T helper cells
NKT cells	Natural killer T cells
MAPK	Mitogen-activated protein kinase
PI3K/Akt	Phosphatidylinositol 3-kinase/Protein kinase B
ECM	Extracellular matrix
